# How hard is it to encapsulate life? The general constraints on encapsulation

**DOI:** 10.1098/rstb.2024.0297

**Published:** 2025-10-02

**Authors:** Christopher P Kempes, Diana Avila, Cole Mathis

**Affiliations:** ^1^ Santa Fe Institute of Science, 1399 Hyde Park Rd, Santa Fe, NM 87501-8943, USA; ^2^ Biodesign Institute, and School of Complex Adaptive Systems, Arizona State University, Tempe, AZ 85281, USA

**Keywords:** space constraints, cell scaling, maximum growth rate

## Abstract

Many studies of the origins of life focus on the advantages of encapsulation. Independent of these advantages, there may be serious challenges to overcome for a system to become encapsulated. Here, we address the general constraints associated with encapsulating a biotic system. We first consider the extant biochemical system of Earth and show that small changes in the rates and sizes of macromolecules can easily make encapsulation difficult. We show that if the ribosome is slower for its size, there is a threshold where modern life could not be encapsulated in cells. We also show how the largest cells are limited by the ribosome rate per size. We are not committed to this system as being universal, but it provides a nice test case where we have complete knowledge of the dynamics and molecular features. To generalize our results, we considered a generic autocatalytic system and a system that has separated informational and functional molecules. In both cases, we find bounds on the allowable growth rates of cells based on the effective rates and volume fractions of cells. We also illustrate how environmental loss rates set the allowable volume fraction of cells as a function of the effective catalytic rate of an autocatalytic set. These results provide a general window into the challenges of encapsulation for any prebiotic or biotic system and are applicable in diverse contexts from the origins of life, to astrobiology, to synthetic biology.

This article is part of the theme issue ‘Origins of life: the possible and the actual’.

## 1. Introduction

Life as we know it on Earth is encapsulated in cellular containers. This ubiquitous encapsulation profoundly shapes our conceptions of what living systems are and how they must be organized. Consequently, encapsulation is widely considered to be a fundamental feature of the origins of life. Encapsulation has been connected to the increased power of selection in an evolutionary dynamic, the concentration of chemicals for enhanced reaction speeds, the protection of a cooperative chemical dynamic from the environment or ‘cheater’ chemicals, and the stability of a reaction network as a far-from-equilibrium system [1–6]. In the most general sense, there could be ways to achieve life-like phenomena without encapsulation, such as with a two-dimensional tiling organism that lives on surfaces (e.g. [7] and [8] for reviews), but our goal here is not to discuss the inevitability of encapsulation. Instead, we are interested in how hard a problem encapsulation is. What are the fundamental constraints on encapsulation, and how do these shape the possibilities for encapsulated life?

Prebiotic chemistry has a long history of investigations into plausible origins of cellular containers, as well as synthetic analogues or alternatives to the phospholipid bilayers used in modern biochemistry [9,10]. There is significant ambiguity in the evolutionary origin of modern phospholipid cell membranes, in part owing to deep divergences between the precise molecules used by bacteria and archaea [11]. However, there is broad agreement that the last universal common ancestor (LUCA) was likely encapsulated in a lipid bilayer. The single molecular components of these containers are found in a variety of simple one-pot prebiotic chemistry experiments and are even found in carbonaceous meteorites [12–14]. These molecules can spontaneously self-assemble into vesicles, even in non-ideal conditions [15]. Vesicles formed from these components vary in volume, with typical radii on the order of 0.1−10.0 µm, with an approximate volume range of $10^{-20}-10^{-14}$ m^3^, which strongly overlaps with the volume range of modern biology.

Synthetic alternatives to lipid bilayer vesicles have been investigated: for example, polyester micro-droplets generated by the drying of α-hydroxy acids [16]. The compartments have typical radii in the range of 10−100s µm, which is slightly high in range compared with known cellular life. Even more speculatively, inorganic containers have been proposed for synthetic cells [17]. These containers are hybrid organic–inorganic membranes made using small molecule organics and poly-oxometalates, with typical radii on the order of 50 µm up to millimetre scales. Given the profound differences in these alternative containers, what consequences might they have for the biochemistries within them? Our goal here is to illustrate how volume considerations alone place constraints on the underlying biochemical systems in encapsulations. We show that a lot can be said about encapsulation without knowledge of biochemical specifics.

Recently, the space constraints of Earth’s simplest organisms, bacteria, archaea and viruses have been elucidated through theory that is verified by measurements. For bacteria, the interconnection of chemical reaction rates, diffusion, macro-molecular volumes and the specific architecture of extant life’s physiology and metabolism (e.g. DNA, ribosomes, and functional proteins) sets constraints on both the largest and smallest cells [18–22]. Similarly, viral capsid size is set by the packing of its hereditary material [23,24].

We begin in §2 by exploring the parameter space around extant life to see how hard it is to encapsulate life. For example, what if life had a much slower ribosome but the same protein concentration? What if the same metabolic process required twice the number of enzymes? What if the volume of folded protein were much larger? We investigate a variety of these contexts and show how encapsulation would be fairly difficult under certain perturbations. We introduce theory that predicts the outcomes of these shifts away from extant life and that reveals the key combination of parameters.

We next generalize our theory in §3 to represent an abstract cell and perform the same analysis. Here, we are able to introduce some universal parameters that predict bounds on encapsulation. These universals would apply to alternative origins of life, life that used radically different molecules, including small molecules and/or macromolecular systems, and life that evolved in much different environments from those on Earth. The theory we develop here is useful for bioengineering and synthetic biology, the reconstruction of possible early life trajectories on Earth, and the search for life beyond Earth.

## 2. Encapsulating slightly different life

Previous models of bacteria have shown that the requirements for different macromolecules can be predicted from cell size [20]. Those models specify the requirements for ribosomes, tRNA and mRNA as a function of protein content and growth rate. Protein concentrations and growth rates are known to scale with cell size for biophysical reasons [22]. Genome size also scales with cell size and introduces constraints on physical space in cells in addition to those for biological rate [25].

We can fully generalize this model by considering the key parameters that might vary in an alternative evolutionary history. This is strongly conditioned on the DNA, RNA, ribosome and protein system specific to life on Earth, which is unlikely to be conserved across the universe. However, slight variations to the extant system illustrate how sensitive cellular possibilities are to small changes in macromolecular properties, and provide insights into the evolutionary pressures faced by life throughout its origin and evolution on Earth.

The previous model derived in [20] specifies that the number of ribosomes, $N_{r}$, follows


(2.1) $$N_{r}\geq \frac{l_{p}N_{p}\left(\frac{\phi }{\mu }+1\right)}{\frac{r_{r}}{\mu }-l_{r}\left(\frac{\eta }{\mu }+1\right)},$$

where μ is growth rate ($\mu =\ln(2)/t_{d}$ given a division time $t_{d}$), $l_{r}$ is the length of all of the ribosomal protein transcripts in base pairs, $l_{p}$ is the length of an average protein, $r_{r}$ (bp s${,}^{-1}$) is the rate at which transcripts are moved through the ribosome, and η (s${,}^{-1}$) and ϕ (s${,}^{-1}$) are degradation rates for ribosomes and proteins, respectively. Since we are interested in encapsulation limits, macromolecular numbers will need to be converted to total volumes throughout our analysis. For each case, we will have that the volume of component i *is* given by $V_{i}=v_{i}N_{i}$ where $v_{i}$ *is* the volume of one molecule. For ribosomes, this is $V_{r}=v_{r}N_{r}$.

As mentioned above the total number of proteins, $N_{p}$, follows a scaling law of cell size given by


(2.2) $$N_{p}=P_{0}V_{c}^{\beta_{p}},$$

which has been explained biophysically [20,22]. The total protein volume in the cell is given by $V_{p}=v_{p}N_{p}$.

Similarly, the growth rate follows


(2.3) $$\mu =\mu_{0}V_{c}^{\beta_{\mu }},$$

which also has a biophysical explanation [19]. The connection to basic biophysics means that the scaling exponents, β, of these relationships are unlikely to change across the universe although the normalization constants, $P_{0}$ and $\mu_{0}$, are likely to change given a wide variety of biochemical and physiological contingencies over evolutionary history. For example, consider a set of enzymes that catalysed the exact same reaction but were all twice as large in volume as the proteins that extant cells use. This difference could be a consequence of different polymer backbones, such as RNA providing enzymatic function. Such a change would alter their diffusivity and affect the value of $P_{0}$ given the arguments in [22]. Understanding the full space of molecules that could catalyse the same reactions is beyond our current capacity and is likely to be effectively innumerable. However, we can treat the abstracted problem by considering shifts to the key parameters in the above model.

**Figure 1.**
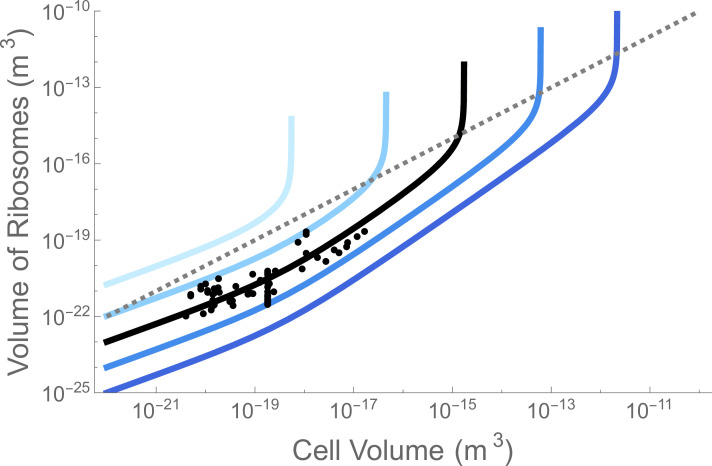
The space requirements of ribosomes within the cell (equation (2.1)). The black points and curve are the ribosome volume in modern cells on Earth (data from [20]). The dashed grey line is the total volume of the cell where it can be seen that ribosomes always exceed this volume for the largest sizes. The blue curves are for different values of the basepair processing rate of the ribosome, going from $10^{-2}\times r_{r}$ (top and light blue) to $10^{2}\times r_{r}$ (bottom and dark blue) in $\log_{10}\;$ steps and where $r_{r}=63$ (bp s^–1^). The plot illustrates that slight modifications to this fundamental biochemical parameter can greatly affect the space requirements for ribosomes. If the ribosome were 100× faster (dark blue) then cells could be ≈1500× larger before reaching the ribosome catastrophe. If the ribosome were 42× slower (light blue), then cells could not be encapsulated at all given the current protein concentrations and growth rates.

Using the previous model [20] described above we can now hold $N_{p}$ and μ to the known values on Earth, which do have biophysical motivations [19,22], and imagine a world where evolution had discovered a different ribosome. One of the key features of the ribosome is its speed, and we can imagine a ribosome that was much faster or slower as given by changes in $r_{r}$ in equation (2.1). Figure 1 shows variations in $r_{r}$ ranging from 100× slower to 100× faster. These small changes in speed limit both the range of possible cell sizes and whether cells can be encapsulated at all. For a ribosome that was 10x slower, the upper bound on cell size—the ‘ribosome catastrophe’ [20]—would decrease by 100-fold. This is a severe change in the possibilities for single-cell life. For a ribosome that is 42× slower, encapsulated life is not possible given the values for protein concentration and growth rates on Earth.

Equation (2.1) predicts that there is a cell size where the required ribosomes will exceed the available space of the cell, which is visible in each curve of figure 1. It is easy to see that such a limit should exist because there is a size for which the denominator of equation (2.1) is equal to 0. This limit is where the required number of ribosomes goes to infinity. To avoid this bound—the ‘ribosome catastrophe’ [20]—it must be the case that


(2.4) $$\begin{array}{lcr} & \mu \leq \frac{r_{r}}{l_{r}}-\eta . & \end{array}$$

Although ribosomes will fully fill the cell just before this catastrophe, this limit sets the critical scale where encapsulation becomes infeasible.

This is a much more compact representation of the bounds related to basic biochemistry that does not require knowledge of the protein concentration of the cell. It is possible to directly calculate the maximum division rate of a cell given $l_{r}$, η, and $r_{r}$, where it is useful to note that the speed per size $r_{r}/l_{r}$ is the key parameter relative to the decay rate. All of this can be translated into cell size by using the scaling for μ given above, which yields


(2.5) $$\begin{array}{lcr} & V_{c}\leq {\left(\frac{r_{r}/l_{r}-\eta }{\mu_{0}}\right)}^{1/\beta_{\mu }}. & \end{array}$$

**Figure 2.**
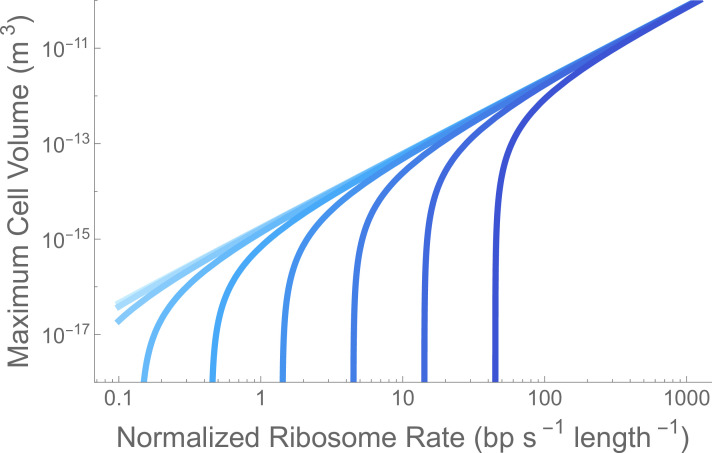
Cell size limitations based on ribosome speed. Curves of the maximum cell volume, $V_{c}$, where the ribosome catastrophe occurs in equation (2.5), as a function of the ribosome speed per size $r_{r}/l_{r}$ (bp s^–1^ length^–1^). Length is the number of base pairs needed to make a ribosome, which tabulates the length of all of the proteins in the ribosome and could be generalized to the total size of any translational machine. The curves are coloured by values of the decay constant η, where light blue is the known value on Earth and the dark blue a decay rate that is $10^{4}$× faster. Each has an asymptote given by $r_{r}/l_{r}=\eta$ that predicts the minimal ribosome rate that allows for encapsulation. This clearly occurs at higher values of the ribosome processing rate $r_{r}/l_{r}$ for increasing decay rates.

Figure 2 shows the maximum cell size, $V_{c}$ against $r_{r}/l_{r}$ for different values of η, which has not been explored previously. Maximum cell size strongly depends on the value of $r_{r}/l_{r}$ for a fixed η and goes to zero for $r_{r}/l_{r}=\eta$. The $r_{r}/l_{r}=\eta$ asymptote is linearly proportional to η, and in the limit of large $r_{r}/l_{r}$ the scaling of maximum cell size converges on $V_{c}\propto {\left(r_{r}/l_{r}\right)}^{1/\beta_{\mu }}$, independent of η. We should expect the largest cells to depend only on the scaling of growth rate, ribosome size and the rate at which ribosomes process transcripts. These ideas of growth rates and catalytic rates should generalize beyond the specific genome-transcription-translation system present on Earth, as we discuss below.

## 3. Generalizing encapsulation

Above we showed that encapsulation can be challenging if certain aspects of our biochemistry had been different. Specifically, it is possible to modify the speed relative to size of the ribosome in such a way that extant biochemistry cannot be encapsulated. This is a proof-of-concept that uses well-known aspects of modern life to show that there may be narrow possibilities for encapsulation. We would now like to generalize these ideas beyond the specifics of Earth’s biochemistry.

### (a) Encapsulating simple autocatalysis

In general, there are many ways to define life [5,26], and also a large number of ways to define cellular or encapsulated life. Here, we focus on an abstraction that is not guaranteed to be universal but that generalizes aspects of cellular life on Earth: an interconnected set of stored information and function that is co-replicated. There are many ways in which life across the universe might differ from this generalization, such as systems with compositional inheritance [27,28]. In such systems, information and function are unitary. That is, all information is simply the current state of the system and how this is divided up into the next generation. There is no special set of molecules with a formalized encoding and decoding of information, as may be likely to evolve at some point in an evolutionary trajectory [6].

One could also add an arbitrary number of translating and interpreting layers. Indeed, it is likely the case that the multi-step information-to-function process that our life uses—DNA to RNA to ribosome to protein—is highly contingent and arbitrary [29]. While not completely general, our abstraction does move away from specific biochemistries and allows for various alternative biochemistries that implement information storage and function.

The most general formulation of cellular biochemistries assumes an autocatalytic set [30]. Such an autocatalytic set could include separated informational (e.g. DNA), translational (e.g. RNA and ribosomes), functional (e.g. proteins) and structural (e.g. lipid membranes) molecules, or these various abstracted functions could be performed by a single class of molecules. For example, imagine a cell with compositional inheritance that simply forms a droplet that is phase-separated from the surrounding fluid. Here, there is no envelope that has distinct molecules from the diverse reactions of metabolism and physiology.

Without committing ourselves to any particular scenario, the most abstracted model of cellular growth given a set of autocatalytic molecules, A, that requires a single limiting resource, R, as an input will have growth dynamics that follow


(3.1) $$\frac{dA}{dt}=\gamma AR,$$

where γ (mol ${,}^{-1}$ m${,}^{3}$ s${,}^{-1}$) is an effective rate of the combined autocatalytic cycle. This generates exponential single cell growth dynamics


(3.2) $$A\left(t\right)=A_{0}e^{\gamma R},$$

and if we consider a cellular fission or division process, which could occur just owing to the size stability of the encapsulation, we have that the time to double in size and divide is


(3.3) $$t_{d}=\frac{\ln(2)}{\gamma R},$$

which is equivalent to a specific population growth rate of μ=γR.

The evolutionary perspective is that the growth rate will be maximized subject to constraints. Our interest here is the challenges associated with encapsulation, and the space constraints on this generalized cell are, very simply, that


(3.4) $$v_{A}A+v_{R}R=1,$$

that is, the combined volume fraction of all of the molecules must sum to one. Here $v_{A}$ and $v_{R}$ are the volumes of molecules (m${,}^{3}$ mol${,}^{-1}$), where this refers to a single molecule for R, and the effective volume of all of the molecules in the combined autocatalytic set for A. It should be noted that A also subsumes the solvent of the cell and anything required for the autocatalytic set to be functional.

Given this constraint, we have that


(3.5) $$R=\frac{1-v_{A}A}{v_{R}},$$

and thus


(3.6) $$\begin{array}{lcr} & \mu =\frac{\gamma \left(1-f_{A}\right)}{v_{R}}. & \end{array}$$

where it is convenient to define $f_{A}=v_{A}A$ as the fraction of cellular volume required by the autocatalytic set. In this model, optimizing the growth rate is clearly about minimizing the space used for the autocatalytic set and maximizing its catalytic rate. It is not a surprising observation that life is benefitted by small catalytically fast molecules, but the model does allow us to immediately set several quantitative constraints on life anywhere.

**Figure 3.**
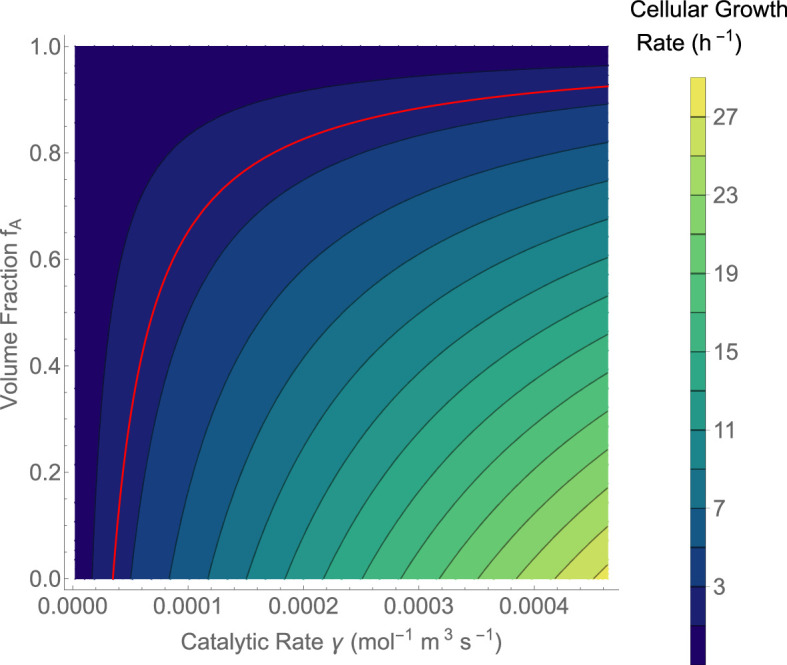
The maximum growth rate of cells based on the effective catalytic rate and the volume fraction of the autocatalytic set. This is the result given in equation (3.6). For reference, the red line is for the growth rate of *E. coli*, where μ≈2 (h^–1^), which is approximately the fastest-growing cell on Earth. This result shows that even for high catalytic rates, the size of the autocatalytic set will dramatically limit growth because of the increased volume fraction associated with more molecules.

First, given that $f_{A}$ and γ can vary under different evolutionary histories—there is a lot of chance in the size and speed of evolved enzymes—we can define the maximum growth rate possible by encapsulated life. Figure 3 provides this growth rate using a reasonable, but broad, range of γ and the entire range of $f_{A}$. The contour that corresponds to the fastest dividing cells on Earth is highlighted, and we observe that small changes to either the required volume packing or effective catalytic rates can easily shift growth rates to be as much as 25 times faster than on Earth. Perhaps the complicated autocatalysis of cellular life on Earth means that it is much slower than it could have been possibly owing to large genetic (DNA) and functional (folded proteins) molecules. This doesn’t consider other tradeoffs beyond space and speed that could explain the system we have on Earth. For example, the genetic system on Earth provides access to a huge catalytic space through folded proteins [5], and our copying, transcribing and translating machineries are exceptionally robust to errors and highly energetically efficient [5]. However, it is interesting to note that it seems feasible to encapsulate life with division rates much quicker than on Earth.

**Figure 4.**
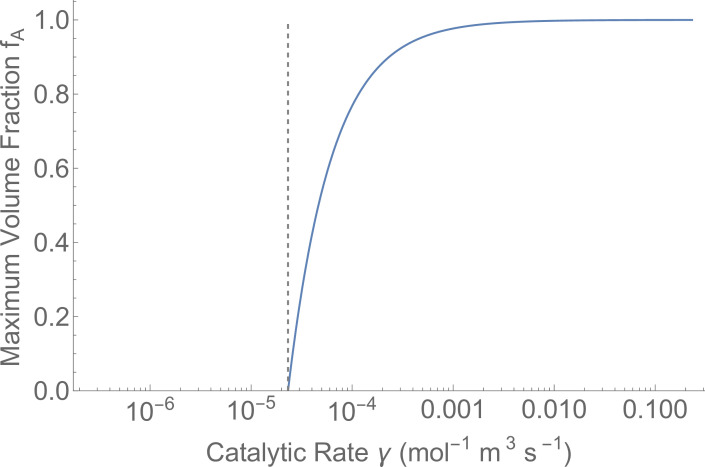
The maximum volume fraction given an effective catalytic rate such that cells escape a particular loss d=0.0004 (s^–1^). The grey dashed line is $dv_{R}$, which sets the feasibility limit according to equation (3.7).

Second, given that all biological systems are characterized by decay and loss—ranging from basic chemical decay processes to cellular sinking, physical destruction, predation and death—it is taken as a given that growth rates must exceed or at least match decay. This can be formalized as μ≥d, where d is the sum of all loss processes as defined by dA/dt=μA−dA, which represents either a homeostatic cell or a chemostat. This condition on μ implies that


(3.7) $$f_{A}\leq 1-d\frac{v_{R}}{\gamma },$$

which immediately limits the molecular sizes and number of molecules in an autocatalytic set given the reaction rates discovered by evolution and situated in an environment with a specific loss rate (figure 4). The surprising aspect of this result is that the allowable volume fraction is set by only the specific loss rate d and the volume $v_{R}$ and catalytic rate γ. There is no dependence on the resource concentration, R, just on the size of the resource molecule that displaces the metabolically active molecules.

This result indicates that as slower catalytic rates are discovered by evolution, the size of the autocatalytic set or the average size of the molecules in that set must be radically reduced. This may seriously limit early evolution to simple encapsulated sets as slow rates are likely to be discovered. We propose that initial encapsulation of rudimentary systems is likely to involve small autocatalytic sets. However, for very high catalytic rates there is a lot of flexibility in the number of molecules and their size as they can nearly fill the cell. Thus, as rates are refined and increased by evolution, the autocatalytic set can increase in size with more complex molecules. If very fast rates are discovered early, which may be unlikely, then it might be possible to encapsulate complicated, large and messy sets early, which could lead to diverse evolutionary trajectories.

### (b) Encapsulating a genetic system

The above model considers all cellular material as part of one autocatalytic cycle without differentiating functional classes of interacting molecules. However, there are many good reasons to separate out classes of molecules. For example, a separated informational molecule may shift the dynamics of selection, increase the information storage fidelity and change the autocatalytic dynamics of the cell [6]. Cells with separate classes of molecules may face different challenges in encapsulating given the dynamics of interacting molecules and because each molecule may be subject to different physical constraints.

We consider the simplest abstraction of a separated genetic system with functional molecules and genetic molecules connected in an autocatalytic loop. The dynamics of this system follow


(3.8)dfdt=γgr1,(3.9)dgdt=αfr2,

where g use resource $r_{1}$ to produce f with rate γ, and f uses resource $r_{2}$ to produce g with rate α.

The time-evolution solutions of these equations lead to a time to divide that follows


(3.10) $$t_{d,g}=\frac{\log\left(\frac{\sqrt{\alpha f_{0}^{\;2}r_{2}+3\gamma g_{0}^{2}r_{1}}+2\sqrt{\gamma }g_{0}\sqrt{r_{1}}}{f_{0}\sqrt{\alpha r_{2}}+g_{0}\sqrt{\gamma r_{1}}}\right)}{\sqrt{\alpha \gamma r_{1}r_{2}}},$$

and


(3.11) $$t_{d,f}=\frac{\log\left(\frac{\sqrt{3\alpha f_{0}^{\;2}r_{2}+\gamma g_{0}^{2}r_{1}}+2\sqrt{\alpha }f_{0}\sqrt{r_{2}}}{f_{0}\sqrt{\alpha r_{2}}+g_{0}\sqrt{\gamma r_{1}}}\right)}{\sqrt{\alpha \gamma r_{1}r_{2}}}$$

where $g_{0}$ and $f_{0}$ are the concentrations at cell birth. For the cell to remain in compositional equilibrium, it must be the case that the two pools double at the same time and $t_{d,g}=t_{d,f}$*,* which implies that


(3.12) $$f_{0}=\frac{g_{0}\sqrt{r_{1}\gamma }}{\sqrt{r_{2}\alpha }}.$$

The space constraints of the system imply that


(3.13) $$v_{f}f+v_{g}g+v_{1}r_{1}+v_{2}r_{2}=1,$$

which gives


(3.14) $$\begin{array}{lcr} & r_{1}=\frac{1-f_{0}v_{f}-g_{0}v_{g}-r_{2}v_{r2}}{v_{r1}}. & \end{array}$$

Taken together, we have that the growth rate is


(3.15) $$\begin{array}{lcr} & \mu =\sqrt{\frac{\alpha \gamma r_{2}\left(1-f_{0}v_{f}-g_{0}v_{g}-r_{2}v_{r2}\right)}{v_{r1}}}. & \end{array}$$

This system clearly has an optimal resource concentration $r_{2}$ that trades off space with the autocatalytic system. This maximum growth rate can be found by considering $d\mu /dr_{2}=0$, which yields


(3.16) $$r_{2,opt}=\frac{1-f_{0}v_{f}-g_{0}v_{g}}{2v_{r2}},$$

and thus


(3.17) $$\mu_{opt}=\frac{1}{2}(1-f_{0}v_{f}-g_{0}v_{g})\sqrt{\frac{\alpha \gamma }{v_{r1}v_{r2}}}.$$

**Figure 5.**
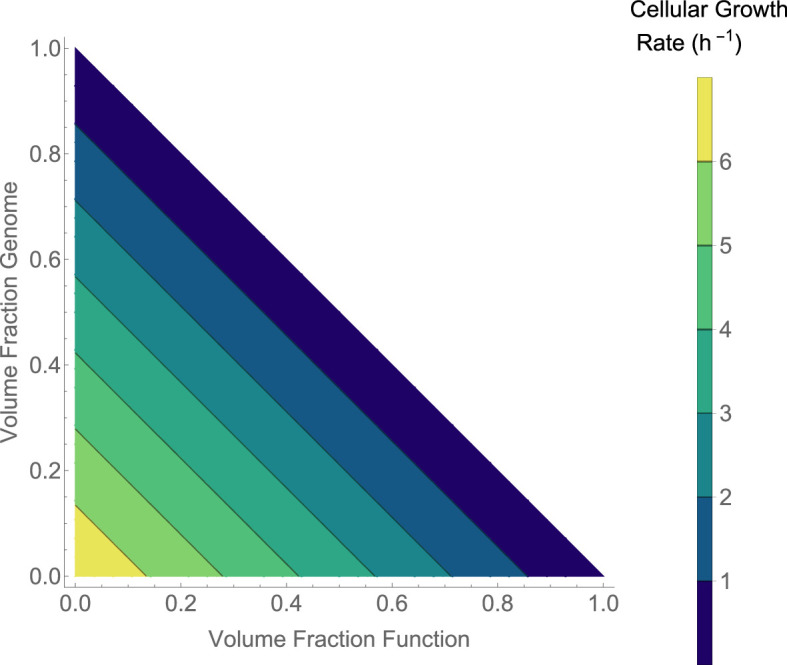
The maximum growth rate based on the volume fractions of genetics and functional molecules. The upper line is the boundary where growth becomes infeasible and represents the maximum volume fraction of genetics based on the volume fraction of functional molecules.

Note that this result is similar to equation (3.6). The added insight is that if either f or g is determined by other constraints of the system, for example diffusive considerations [22], we can predict the other based on space constraints (figure 5).

## 4. Discussion

Our results indicate a critical limit on cell size imposed by the speed and stability of ribosomes. If we anticipate that nascent life was limited to less efficient ribosomes (normalized to the number of encoded genes), or to less stable ribosomes that degraded faster, this would imply stricter upper limits on cell sizes. For example, if we knew that nascent ribosomes decayed 100× faster than modern ones, and that they operated 10× more slowly than their modern counterparts, then encapsulation in self-assembled lipid vesicles would be impossible because the vesicles’ typical size (1 micron radii, $10^{-18}$ m^3^) would be too large. Trade-offs of this nature provide constraints that can inform and limit divergent aspects of origin of life research, enabling scientists to piece together different parts of the puzzle [31].

Interestingly, evidence suggests that for lipid vesicles, their size and stability may be dependent on geochemical conditions [15,32]. This combination of geochemical parameters controlling self-assembly and biophysical constraints limiting viable cell sizes suggests that the results presented here could be used to significantly limit the search space for the origin of life [33].

Synthetic evolutionary experiments based on encapsulated but unconstrained (e.g. non-enzymatic) autocatalytic sets are now being explored [34]. These experiments demonstrate that differential rates among containers can be interpreted as a form of fitness, even in the absence of genetic storage, translation machinery or functional macromolecules. Interestingly, consistent with equation (3.7), these dynamics are driven by a fast, short-loop autocatalytic cycle: the formose reaction [35].

In all of our results, the volume fraction of the autocatalytic set decreases growth rate because it displaces the space available for the consumed resource. In general, we should expect that it is increasingly difficult to encapsulate autocatalytic sets with bigger molecules or autocatalytic sets with greater numbers of components. This problem is particularly relevant if the catalytic rates are slow. If there was not much capacity for selection and evolution in the environment before the evolution of cells, then we should expect that the catalytic rate was relatively unrefined by evolution, and early life was forced to encapsulate small autocatalytic sets. However, if there were avenues for strong refinement of rates within the environment, then early life could encapsulate relatively large and complex autocatalytic sets. These distinctions point to the need for theories of pregenetic evolution, such as assembly theory [36], compositional inheritance [27,28] and multilevel selection [1,3], that constrain how much selection we should expect in different chemical spaces.

The volume constraints highlighted here are only one type of physical constraint and many others affect cells. For example, encapsulations are also limited by the physics of self-assembly [37]. Future work should focus on how rate, space, functional, self-assembly, diffusive and replicative constraints all determine the feasibility of encapsulated life. Here, there is a rich set of phenomena to explore, such as the interconnection of greater numbers of molecule types, the structure of autocatalytic networks [38] and the scaling behaviour of enzyme diversity with encapsulation size [39,40].

## Data Availability

We included a previously published data compilation for comparison in one figure with a citation of that compilation.

## References

[1] Szathmáry E, Demeter L. 1987 Group selection of early replicators and the origin of life. J. Theor. Biol. **128**, 463–486. (10.1016/S0022-5193(87)80191-1) 2451771

[2] Saha R, Pohorille A, Chen IA. 2014 Molecular crowding and early evolution. Orig. Life Evol. Biospheres **44**, 319–324. (10.1007/s11084-014-9392-3) 25585804

[3] Takeuchi N, Hogeweg P. 2009 Multilevel selection in models of prebiotic evolution II: a direct comparison of compartmentalization and spatial self-organization. PLoS Comput. Biol. **5**, e1000542. (10.1371/journal.pcbi.1000542) 19834556 PMC2757730

[4] Lane N, Martin WF. 2012 The origin of membrane bioenergetics. Cell **151**, 1406–1416. (10.1016/j.cell.2012.11.050) 23260134

[5] Smith E, Morowitz HJ. 2016 The origin and nature of life on earth: the emergence of the fourth geosphere. Cambridge, UK: Cambridge University Press. (10.1017/CBO9781316348772)

[6] Solé R *et al*. 2024 Fundamental constraints to the logic of living systems. Interface Focus **14**, 495–500. (10.1098/rsfs.2024.0010) PMC11503024 39464646

[7] Wächtershäuser G. 1988 Before enzymes and templates: theory of surface metabolism. Microbiol. Rev. **52**, 452–484. (10.1128/mr.52.4.452-484.1988) 3070320 PMC373159

[8] Plum AM, Baum DA. 2022 Aces in spaces: autocatalytic chemical ecosystems in spatial settings. arXiv 2212.14445. (10.48550/arXiv.2212.14445)

[9] Oparin AI. 1953 The origin of life. New York, NY: Dover Publications.

[10] Lai YC, Chen IA. 2020 Protocells. Curr. Biol. **30**, R482–R485. (10.1016/j.cub.2020.03.038) 32428486

[11] Lombard J, López-García P, Moreira D. 2012 The early evolution of lipid membranes and the three domains of life. Nat. Rev. Microbiol. **10**, 507–515. (10.1038/nrmicro2815) 22683881

[12] McCollom TM, Ritter G, Simoneit BRT. 1999 Lipid synthesis under hydrothermal conditions by Fischer-Tropsch-type reactions. Orig. Life Evol. Biosph. **29**, 153–166. (10.1023/A:1006592502746) 10227201

[13] Scherer S, Wollrab E, Codutti L, Carlomagno T, da Costa SG, Volkmer A, Bronja A, Schmitz OJ, Ott A. 2017 Chemical analysis of a ‘Miller-type’ complex prebiotic broth. Part II: gas, oil, water and the oil/water-interface. Orig. Life Evol. Biosph. **47**, 381–403. (10.1007/s11084-016-9528-8) 27896547 PMC5705758

[14] Deamer DW. 1985 Boundary structures are formed by organic components of the Murchison carbonaceous chondrite. Nature **317**, 792–794. (10.1038/317792a0)

[15] Jordan SF, Rammu H, Zheludev IN, Hartley AM, Maréchal A, Lane N. 2019 Promotion of protocell self-assembly from mixed amphiphiles at the origin of life. Nat. Ecol. Evol. **3**, 1705–1714. (10.1038/s41559-019-1015-y) 31686020

[16] Jia TZ, Chandru K, Hongo Y, Afrin R, Usui T, Myojo K, Cleaves HJ II. 2019 Membraneless polyester microdroplets as primordial compartments at the origins of life. Proc. Natl Acad. Sci. USA **116**, 15830–15835. (10.1073/pnas.1902336116) 31332006 PMC6690027

[17] Cooper GJT, Kitson PJ, Winter R, Zagnoni M, Long D, Cronin L. 2011 Modular redox‐active inorganic chemical cells: iCHELLs. Angew. Chem. **123**, 10557–10560. (10.1002/ange.201105068) 21901807

[18] DeLong JP, Okie JG, Moses ME, Sibly RM, Brown JH. 2010 Shifts in metabolic scaling, production, and efficiency across major evolutionary transitions of life. Proc. Natl Acad. Sci. USA **107**, 12941–12945. (10.1073/pnas.1007783107) 20616006 PMC2919978

[19] Kempes CP, Dutkiewicz S, Follows MJ. 2012 Growth, metabolic partitioning, and the size of microorganisms. Proc. Natl Acad. Sci. USA **109**, 495–500. (10.1073/pnas.1115585109) 22203990 PMC3258615

[20] Kempes CP, Wang L, Amend JP, Doyle J, Hoehler T. 2016 Evolutionary tradeoffs in cellular composition across diverse bacteria. ISME J. **10**, 2145–2157. (10.1038/ismej.2016.21) 27046336 PMC4989312

[21] Kempes CP, van Bodegom PM, Wolpert D, Libby E, Amend J, Hoehler T. 2017 Drivers of bacterial maintenance and minimal energy requirements. Front. Microbiol. **8**, 31. (10.3389/fmicb.2017.00031) 28197128 PMC5281582

[22] Ritchie ME, Kempes CP. 2023 Metabolic scaling in small life forms. bioRxiv 2023.12.20.572702. (10.1101/2023.12.20.572702)

[23] Jover LF, Effler TC, Buchan A, Wilhelm SW, Weitz JS. 2014 The elemental composition of virus particles: implications for marine biogeochemical cycles. Nat. Rev. Microbiol. **12**, 519–528. (10.1038/nrmicro3289) 24931044

[24] Requião RD, Carneiro RL, Moreira MH, Ribeiro-Alves M, Rossetto S, Palhano FL, Domitrovic T. 2020 Viruses with different genome types adopt a similar strategy to pack nucleic acids based on positively charged protein domains. Sci. Rep. **10**, 5470. (10.1038/s41598-020-62328-w) 32214181 PMC7096446

[25] Lynch M, Trickovic B, Kempes CP. 2022 Evolutionary scaling of maximum growth rate with organism size. Sci. Rep. **12**, 22586. (10.1038/s41598-022-23626-7) 36585440 PMC9803686

[26] Benner SA. 2010 Defining life. Astrobiology **10**, 1021–1030. (10.1089/ast.2010.0524) 21162682 PMC3005285

[27] Segré D, Ben-Eli D, Lancet D. 2000 Compositional genomes: prebiotic information transfer in mutually catalytic noncovalent assemblies. Proc. Natl Acad. Sci. USA **97**, 4112–4117. (10.1073/pnas.97.8.4112) 10760281 PMC18166

[28] Wu M, Higgs PG. 2008 Compositional inheritance: comparison of self-assembly and catalysis. Orig. Life Evol. Biospheres **38**, 399–418. (10.1007/s11084-008-9143-4) 18636340

[29] Adamala K, Szostak JW. 2013 Competition between model protocells driven by an encapsulated catalyst. Nat. Chem. **5**, 495–501. (10.1038/nchem.1650) 23695631 PMC4041014

[30] Rasmussen S. 1989 Toward a quantitative theory of the origin of life. In Artificial life (ed. C Langton), pp. 79–104. New York, NY: Addison-Wesley Publishing Company, Inc.

[31] Lane N, Xavier JC. 2024 To unravel the origin of life, treat findings as pieces of a bigger puzzle. Nature **626**, 948–951. (10.1038/d41586-024-00544-4) 38409541

[32] Jordan SF, Nee E, Lane N. 2019 Isoprenoids enhance the stability of fatty acid membranes at the emergence of life potentially leading to an early lipid divide. Interface Focus **9**, 20190067. (10.1098/rsfs.2019.0067) 31641436 PMC6802135

[33] Foote S, Sinhadc P, Mathis C, Walker SI. 2023 False positives and the challenge of testing the alien hypothesis. Astrobiology **23**, 1189–1201. (10.1089/ast.2023.0005) 37962842

[34] Lu H *et al*. 2024 Small-molecule autocatalysis drives compartment growth, competition and reproduction. Nat. Chem. **16**, 70–78. (10.1038/s41557-023-01276-0) 37550391

[35] Huskey WP, Epstein IR. 1989 Autocatalysis and apparent bistability in the formose reaction. J. Am. Chem. Soc **111**, 3157–3163. (10.1021/ja00191a008)

[36] Sharma A, Czégel D, Lachmann M, Kempes CP, Walker SI, Cronin L. 2023 Assembly theory explains and quantifies selection and evolution. Nature **622**, 321–328. (10.1038/s41586-023-06600-9) 37794189 PMC10567559

[37] Solé RV. 2009 Evolution and self-assembly of protocells. Int. J. Biochem. Cell Biol. **41**, 274–284. (10.1016/j.biocel.2008.10.004) 18951997

[38] Blokhuis A, Lacoste D, Nghe P. 2020 Universal motifs and the diversity of autocatalytic systems. Proc. Natl Acad. Sci. USA **117**, 25230–25236. (10.1073/pnas.2013527117) 32989134 PMC7568248

[39] Gagler DC, Karas B, Kempes CP, Malloy J, Mierzejewski V, Goldman AD, Kim H, Walker SI. 2022 Scaling laws in enzyme function reveal a new kind of biochemical universality. Proc. Natl Acad. Sci. USA **119**, e2106655119. (10.1073/pnas.2106655119) 35217602 PMC8892295

[40] Gondhalekar R, Kempes CP, McGlynn SE. 2023 Scaling of protein function across the tree of life. Genome Biol. Evol. **15**, d214. (10.1093/gbe/evad214) PMC10715193 38007693

